# Patient’s experience with subcutaneous and oral methotrexate for the treatment of rheumatoid arthritis

**DOI:** 10.1186/s12891-016-1254-x

**Published:** 2016-09-26

**Authors:** J. R. Curtis, F. Xie, D. Mackey, N. Gerber, A. Bharat, T. Beukelman, K. G. Saag, L. Chen, B. Nowell, S. Ginsberg

**Affiliations:** 1 University of Alabama at Birmingham, 510 20th Street South, FOT 802, Birmingham, AL 35294 USA; 2 Global Healthy Living Foundation, Upper Nyack, NY 10960 USA

**Keywords:** Rheumatoid arthritis, Methotrexate, Adherence, Side effects

## Abstract

**Background:**

Despite the prominent position of methotrexate (MTX) in Rheumatoid Arthiris (RA) therapeutics, its real-world effectiveness may be influenced by a relative lack of tolerability or other side effects that physicians may not be aware of but that are bothersome to patients.

The aim of this study is to identify suboptimal patient experience with MTX and to raise awareness for clinicians to identify opportunities to mitigate bothersome symptoms and side effects and optimize response to MTX.

**Methods:**

We conducted a prospective, cross-sectional, online survey among RA patients who were members of Creakyjoints, a large arthritis patient community. Eligible participants must have recently initiated a new biologic, subcutaneous (SQ) MTX, or oral MTX in the last 12 months and were uniquely assigned to one of these 3 groups. Descriptive statistics were used to compare patient-reported side effects and tolerability related to MTX use in the 3 medication groups (SQ MTX, oral MTX, and biologic).

**Results:**

A total of 382 (85 %) of 448 eligible patients completed the survey and were grouped as: biologic (*n* = 218), SQ MTX (*n* = 49), and oral MTX (*n* = 115). Demographics were mean standard deviation (SD) age 48 (10) years, 92 % white, 91 % women. Symptoms significantly more prevalent in the SQ and oral MTX groups included diarrhea, fatigue, malaise, and hair loss. Injection related pain was lower with SQ MTX compared to SQ biologics. Out of a total of 8 potential symptoms and side effects examined, higher dose MTX (> = 20 mg/week) was associated with a 2.26 (1.25–4.09) greater likelihood of more side effects referent to < =10 mg/week.

**Conclusion:**

Results from this real-world RA patient cohort suggest that MTX is accompanied by many patient-reported side effects and tolerability problems that may be under-recognized by physicians. These may impact both treatment satisfaction and medication adherence.

## Background

Methotrexate (MTX) has been a valuable treatment for rheumatoid arthritis (RA) for more than 30 years, and it remains the anchor drug for RA therapy [1–3]. Although MTX is generally safe, intolerability may be an important cause of treatment discontinuation and often includes gastrointestinal (GI) adverse events (AEs) [4–6]. While most patients with RA in the United States receive MTX orally [7], subcutaneous (SQ) administration is available and may provide advantages in both efficacy and tolerability [8–12].

Compared to oral MTX, SQ MTX has been shown to provide increased bioavailability, which may confer greater efficacy, particularly at higher doses in which oral MTX bioavailability appears to plateau [11, 13]. SQ MTX has been associated with a reduced frequency and intensity of some GI AEs compared with oral MTX [14, 15] which may improve treatment compliance and reduce MTX discontinuation rates. However, the prevalence of symptoms and side effects associated with oral and SQ MTX from data other than clinical trials has been characterized in only a very limited fashion. In randomized controlled trials (RCTs), for example, patients may report more severe side effects as adverse events, yet fail to mention milder yet meaningfully bothersome side effects (e.g. injection site pain) because they are not systematically queried [16–18].

Given a limited experience from real-world settings to assess the prevalence of patient-reported symptoms and AEs that are associated with MTX or biologics, this study evaluated these outcomes in RA patients who initiated oral MTX, SQ MTX, and biologic therapies. The goal was to identify suboptimal patient experience with MTX and to raise awareness for clinicians to identify opportunities to mitigate bothersome symptoms and side effects and optimize response to MTX.

## Methods

### Cohort selection and eligibility

Patient data were collected through a compensated ($25) online survey disseminated by CreakyJoints®, a large arthritis patient community. Recruitment was initiated on March 31, 2014, and ended on November 16, 2014. Eligible patients had self-reported RA and recently (within the last 12 months) initiated a new biologic therapy, SQ MTX, or oral MTX. Patients were uniquely assigned to 1 of 3 groups according to the following hierarchy: biologic or novel small molecule (tofacitinib) (referred to hereafter as the “biologic” arm), SQ MTX, and oral MTX. To avoid selection bias, patients did not need to continue MTX to be eligible for the survey and included in the two MTX arms; for this reason, not all patients were current MTX users at the time of the survey, and therefore reported their experienced based upon their past use of MTX. Patients who initiated (for example) both a biologic and MTX within the past 12 months were assigned to the biologic cohort. All patients were asked to report symptoms experienced in the relevant time frame, irrespective of perceived causality from any particular medication. Patients provided explicit informed consent to participate, and the study was governed by the local University Institutional Review Board at UAB.

### Statistical analysis

The list of patient symptoms AEs were selected based on content knowledge, with a focus on those perceived to be common among oral or SQ MTX users. Similar questions were asked of patients in the biologic group to provide comparability. Patient characteristics by treatment group and frequency of patient-reported AEs were compared using descriptive statistics (chi square and t-tests). The severity of symptoms was measured on an ordinal scale and evaluated using the Cochran-Mantel-Haenszel test for whether the row mean scores differed. The analysis was done twice, the first time representing patients who said that they did not experience the symptom as a “no” response. The second method removed patients who said that they did not experience the symptom and assessed the severity of the symptom conditional on the patient having experienced it. Among all patients who reported that they were taking MTX (including those taking concomitant biologics), the incidence of 8 potentially MTX-associated adverse events was evaluated as an ordinal outcome (0-8 scale) and analyzed using ordinal logistic regression with MTX dose as the independent variable, to test the hypothesis that the number of symptoms was associated with higher MTX dose. Pain associated with the administration of SQ MTX and biologics was reported on a scale from 0 to 10, and mean scores were compared between patients who received etanercept, adalimumab, and SQ MTX. Data were analyzed as complete cases only; those who discontinued the survey prematurely (14.8 % of those eligible) were not analyzed. All analyses were conducted in SAS 9.4.

## Results

**Fig. 1:**
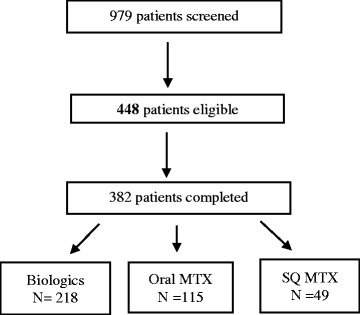
Patient flow chart

Of 979 patients screened for the study, 448 (45.7 %) were eligible and of these, 382 (85.2 %) completed the survey in its entirety (Fig. 1). Based upon medications initiated in the preceding 12 months, patients were uniquely assigned to biologic therapy (*n* = 218), SQ MTX (*n* = 49), or oral MTX (*n* = 115). Their characteristics are described in Table 1. In the overall cohort, mean (SD) age was 48.03 (10.21) years, 91 % were women, and most (92 %) were White and from the U.S (90 %). Approximately 1/3 of the sample self-reported being disabled. Sixty percent of patients were commercially insured. For all 3 treatment groups, the median monthly drug copay for biologics and MTX was less than $25. Demographics and other characteristics were similar between the 3 treatment groups.

**Table 1:** Patient demographics

	Overall	Biologic therapies	SQ MTX	Oral MTX	*P*-value
	*N* = 382	*n* = 218	*n* = 49	*n* = 115	
Age, mean (SD), y	48.03 (10.21)	46.98 (10.44)	49.46 (9.94)	49.43 (9.72)	0.67
Gender, % female	91.10	89.45	89.80	94.78	0.25
Race of participants^a^					
- American Indian	5 (1.3)	4 (1.8)	0 (0.0)	1 (0.9)	0.60
- Alaska Native	1 (0.3)	1 (0.5)	0 (0.0)	0 (0.0)	
- Asian	3 (0.8)	1 (0.5)	1 (2.0)	1 (0.9)	
- Native Hawaiian/other Pacific Islander	1 (0.3)	1 (0.5)	0 (0.0)	0 (0.0)	
- Black/African-American	7 (1.8)	6 (2.8)	1 (2.0)	0 (0.0)	
- White	353 (92.4)	200 (91.7)	45 (91.8)	108 (93.9)	
- Other	12 (3.1)	5 (2.3)	2 (4.1)	5 (4.4)	
Ethnicity					0.17
- Hispanic or Latino	19 (5.0)	8 (3.7)	5 (10.2)	6 (5.2)	
- Not Hispanic or Latino	360 (94.2)	209 (95.9)	43 (87.8)	108 (93.9)	
- No answer	3 (0.8)	1 (0.5)	1 (2.0)	1 (0.9)	
Participants Reside in the United States	343 (89.8)	197 (90.4)	43 (87.8)	103 (89.6)	0.85
Participants not from US	39 (10.2)	21 (9.6)	6 (12.2)	12 (10.4)	
Participant employment status					
- Full time	145 (38.0)	89 (40.8)	14 (28.6)	42 (36.5)	0.09
- Part time	40 (10.5)	18 (8.3)	9 (18.4)	13 (11.3)	
- Retired	25 (6.5)	10 (4.6)	2 (4.1)	13 (11.3)	
- Homemaker	37 (9.7)	18 (8.3)	4 (8.2)	15 (13.0)	
- Student	12 (3.1)	8 (3.7)	1 (2.0)	3 (2.6)	
- Disabled	123 (32.2)	75 (34.4)	19 (38.8)	29 (25.2)	
Participant health insurance					
- Medicare	52 (13.6)	29 (13.3)	7 (14.3)	16 (13.9)	0.54
- Medicare advantage	18 (4.7)	10 (4.6)	3 (6.1)	5 (4.4)	
- Medicaid	28 (7.3)	18 (8.3)	0 (0.0)	10 (8.7)	
- Tri-care/Military	5 (1.3)	5 (2.3)	0 (0.0)	0 (0.0)	
- Commercial/Private	227 (59.4)	134 (61.5)	27 (55.1)	66 (57.4)	
- Other/type	54 (14.1)	24 (11.0)	9 (18.4)	21 (18.3)	
- None	34 (8.9)	20 (9.2)	6 (12.2)	8 (7.0)	
Monthly co-payment (*n*)	N/A				
$0		47 (21.6)	2 (4.1)	15 (13.0)	<0.0001
> $0–25		65 (29.8)	24 (49.0)	68 (59.1)	
$25–$40		13 (6.0)	5 (10.2)	16 (13.9)	
$40–80		23 (10.6)	0 (0.0)	6 (5.2)	
$80–100		7 (3.2)	0 (0.0)	0 (0.0)	
> $100		35 (16.1)	1 (2.1)	0 (0.0)	
Don’t know		0 (0.0)	3 (6.1)	10 (8.7)	
Missing		0 (0.0)	14 (28.6)	0 (0.0)	

data are shown as *n* (%)

*MTX* methotrexate, *SC* subcutaneous, *SD* standard deviation

^a^race data are not mutually exclusive

As shown in Table 2, among the patients treated with a biologic, the most commonly used agents were etanercept (23 %) and adalimumab (24 %). Approximately 2/3 of the biologic users were concomitantly taking MTX, and most of them reported good adherence, having taken all 4 doses in the last 4 weeks. In the MTX arms, 28.6 % of SQ MTX users and 7.8 % of oral MTX users reported that they were not still taking MTX.

**Table 2:** Characteristics of patients that initiated biologics, oral and SQ MTX in the previous 12 months^a^

	Biologic arm	SQ MTX	Oral MTX
	*N* = 218	*N* = 49	*N* = 115
Biologic or novel small molecule initiated		n/a	n/a
Tocilizumab	13 (6.0)		
Certolizumab	11 (5.1)		
Etanercept	51 (23.4)		
Adalimumab	53 (24.3)		
Abatacept	33 (15.1)		
Infliximab	22 (10.1)		
Rituximab	11 (5.1)		
Golimumab	13 (6.0)		
Tofacitinib	11 (5.1)		
Frequency of MTX doses taken in last 4 weeks			
4 doses	133 (61.0)	31 (63.3)	90 (78.3)
3 doses	7 (3.2)	1 (2.0)	9 (7.8)
2 doses	10 (4.6)	1 (2.0)	5 (4.4)
1 dose	1 (0.5)	2 (4.1)	2 (1.7)
0 doses^b^	66 (30.3)	14 (28.6)	9 (7.8)
Not sure	1 (0.5)	0 (0.0)	0 (0.0)
Current MTX dose/week (mg)			
None	95 (43.5)	14 (28.6)	9 (8.0)
Unknown	9 (4.1)	3 (6.1)	5 (4.4)
< = 10	36 (16.5)	8 (16.3)	22 (19.1)
> 10– < 20	42 (19.3)	7 (14.3)	44 (38.3)
> =20	28 (12.8)	17 (34.7)	44 (38.3)
Reasons that patients missed 1 or more MTX doses in the last 4 weeks^c^	*N/A*		
Forgot		1 (2.0)	0 (0.0)
Side effects		2 (4.1)	1 (0.9)
Pharmacy shortage		1 (2.0)	0 (0.0)
Doing well		1 (2.0)	1 (0.9)
Couldn’t afford		1 (2.0)	1 (0.9)
Other reasons		2 (4.1)	4 (3.5)
Willingness to pay for MTX	*N/A*		
$0		1 (2.0)	4 (3.5)
$10		7 (14.3)	13 (11.3)
Up to $25		19 (38.8)	34 (29.6)
$25–$40		5 (10.2)	26 (22.6)
$40–$80		2 (4.1)	6 (5.2)
Did injections hurt?			*N/A*
No, never	55 (25.2)	11 (22.4)	
Yes, rarely	34 (15.6)	14 (28.6)	
Yes, sometime	76 (34.9)	15 (30.6)	
Yes, often	53 (24.3)	9 (18.4)	

Data shown as *n* (%)

*n/a* not asked

^a^to avoid selection bias, patients did not need to continue MTX to be included in the two MTX arms of the survey; for this reason, not all patients are current users

^b^These are patients who reported that they were not currently taking MTX; they had discontinued at the time of the survey. However, some had recently discontinued and thus the counts are not synonymous with the “Current Dose = none” response

^c^response among people who said they took only 1, 2, or 3 doses of MTX in the last 4 weeks

Table 3 describes the frequency of side effects associated with biologics (stratified by concomitant methotrexate use or not), SQ and oral MTX. Overall, the most commonly reported AEs included diarrhea, fatigue, malaise, mental fog, infection, and pain associated with injection. Significant differences were found between the groups in the reported incidence of diarrhea, fatigue, malaise, mental fog, hair loss, and pain associated with the injection. There were no significant differences between the two biologic subgroups (with and without MTX) for any of the symptoms listed. Regarding injection-related pain, 24 % of biologic treated patients said that their injections hurt ‘often’ as compared to 18 % of SQ MTX users [not shown].

**Table 3:** Patient-reported adverse events associated with biologic therapies (with and without methotrexate), subcutaneous methotrexate, and oral methotrexate

AE, *n* (%)	Biologics without MTX	Biologics with MTX	SQ MTX	Oral MTX	*P* value
	*n* = 95	*N* = 123	*n* = 49	*n* = 115	
Hair loss	10 (11)	11 (9)	17 (35)	35 (30)	<0.0001
Diarrhea	2 (2)	6 (5)	6 (12)	26 (23)	<0.0001
Vomiting	2 (2)	6 (5)	4 (8)	8 (7)	0.32
Nausea	11 (12)	17 (14)	27 (55)	39 (34)	<0.0001
Other stomach problems	9 (10)	5 (4)	3 (6)	7 (6)	0.44
Fatigue	21 (22)	27 (22)	36 (73)	64 (56)	<0.0001
Malaise	17 (18)	14 (11)	23 (47)	38 (33)	<0.0001
Mental fog	14 (15)	19 (15)	18 (37)	33 (29)	0.0016
Infection	12 (13)	11 (9)	4 (8)	5 (4)	0.19
Any pain with injections	48^a^ (89)	62^a^ (84)	27 (55)	N/A	<0.0001
Side effect sum^b^					<0.0001
0	67 (71)	91 (74)	5 (10)	36 (31)	
1	4 (4)	7 (6)	9 (18)	12 (10)	
2	4 (4)	7 (6)	7 (14)	19 (17)	
3	10 (11)	5 (4)	12 (24)	17 (15)	
4	6 (6)	6 (5)	8 (16)	14 (12)	
> =5	4 (4)	7 (6)	8 (16)	17 (15)	

*P* values reported from chi-square test

*AE* adverse event, *MTX* methotrexate, *SQ* subcutaneous

^a^Of 128 patients who received biologic therapies subcutaneously

^b^number of side effect of hair loss, diarrhea, vomiting, nausea, other stomach problems, fatigue, malaise, mental fog

**Fig. 2:**
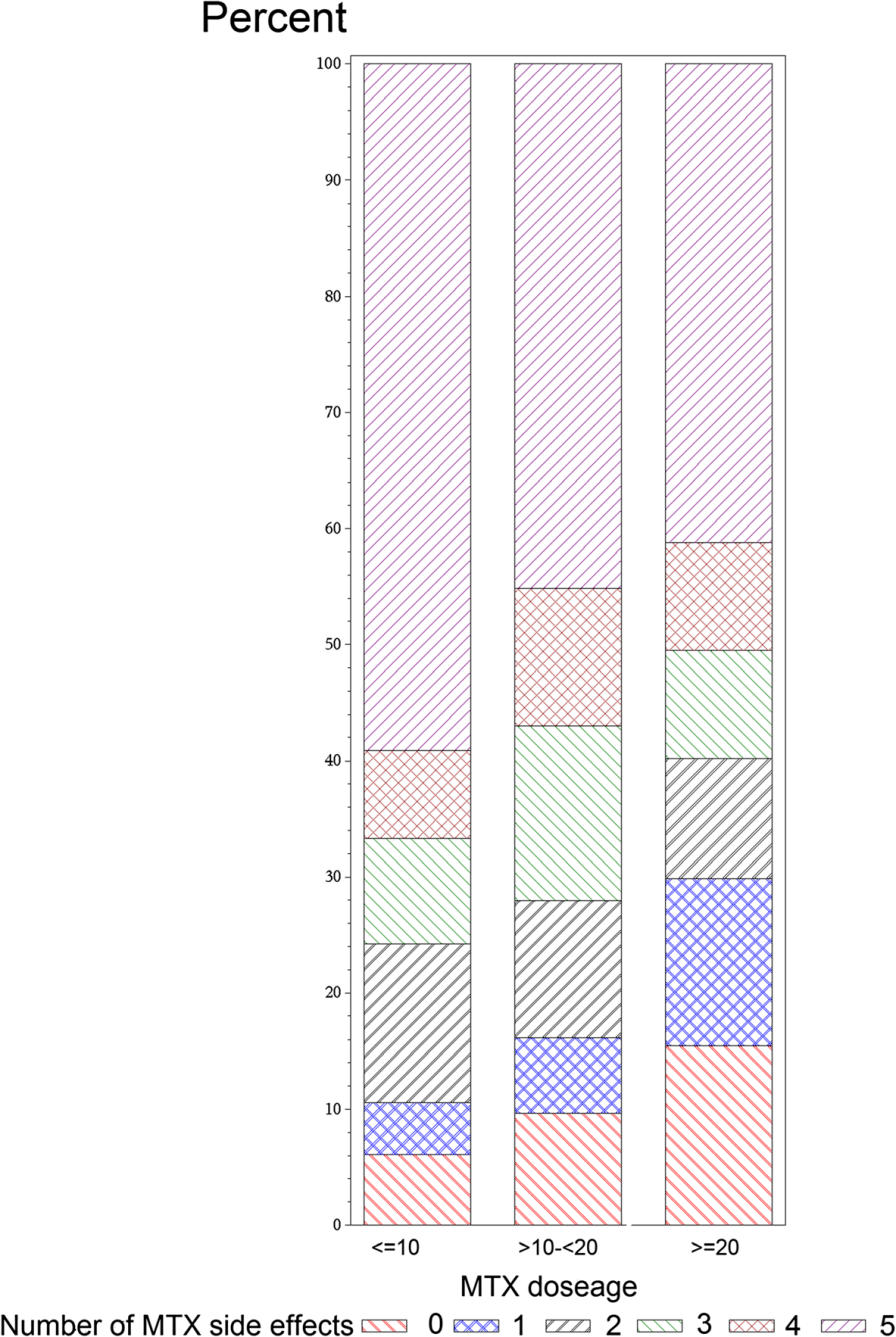
Number of side effects* associated with current use of methotrexate, by dose. *Out of a maximum of 8 possible, as described in the first 8 rows of Table 3. Note: Analysis was restricted to patients who reported current MTX use and known dose (as described in Table 2)

**Fig. 3:**
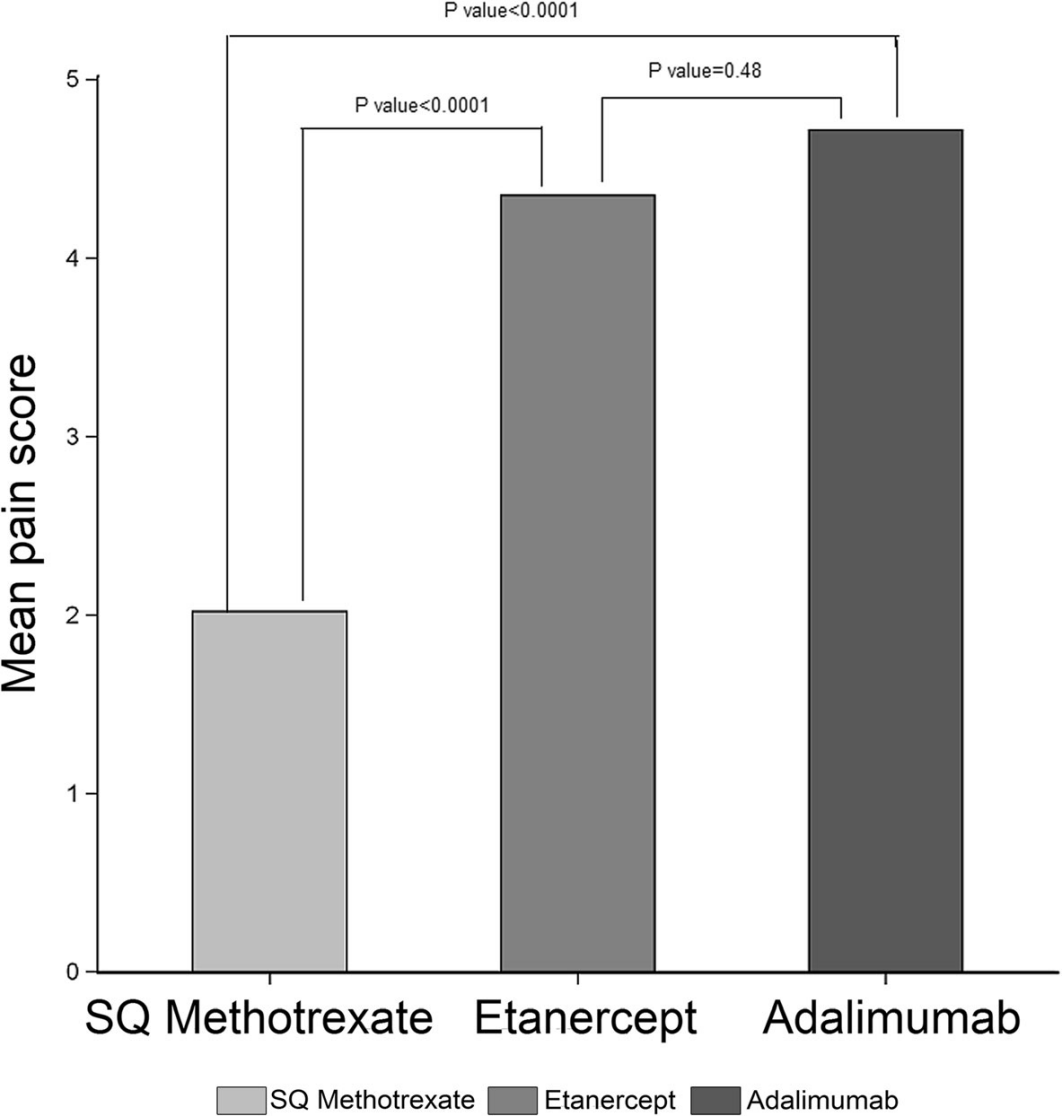
Mean pain scores associated with subcutaneous MTX vs etanercept and adalimumab. *patients who said that they experienced no pain with injection are included as having a 0 pain score. MTX, methotrexate

The severity of each of these patient-reported symptoms is described in Table 4. Differences between the 3 treatment groups largely mirrored the differences in incidence described in Table 3. Conditional on patients having each of these side effects, the severity of the specific side effect did not differ between treatments except for mental fog for which severity was greater in both MTX treatment groups compared to biologics. Among the subgroup of patients currently receiving oral or SQ MTX (with or without biologics), there was an association between the dose of MTX and the number of side effects experienced (Fig. 2). Patients receiving MTX at weekly doses of < = 10 mg had fewer side effects than those receiving higher doses. Results from the ordinal logistic regression showed that referent to < =10 mg/week, patients receiving between 10 and 20 mg/week had a 1.57 (0.86–2.87) higher odds of a higher number of side effects, and patients receiving > =20 mg/week had a 2.26 (1.25–4.09) higher odds of more side effects. Patient-reported pain associated with SQ injections (Fig. 3) was significantly greater for the administration of etanercept and adalimumab compared with SQ MTX (*P* < 0.0001 for each), but etanercept and adalimumab were not different from other another.

**Table 4:** Patient-reported severity of adverse events associated with biologic therapies, subcutaneous methotrexate, and oral methotrexate

		Severity of hair loss						
Therapy, *n* (%)	*N*	No	Trivial	Mild	Moderate	Marked	Severe	So severe I had to stop
Biologic therapies	218	197 (90)	2 (1)	4 (2)	12 (6)	0 (0)	3 (1)	0 (0)
SQ MTX	49	32 (65)	1 (2)	9 (18)	2 (4)	5 (10)	0 (0)	0 (0)
Oral MTX	115	80 (70)	2 (2)	10 (9)	8 (7)	13 (11)	2 (2)	0 (0)
*P* value for row mean score < 0.0001								
*P* value for row mean score differs (excluding “No” responses) = 0.37								
		Severity of diarrhea						
Therapy, *n* (%)	*N*	No	Trivial	Mild	Moderate	Marked	Severe	So severe I had to stop
Biologic therapies	218	210 (96)	1 (.5)	1 (.5)	3 (1.5)	1 (.5)	2 (1)	0 (0)
SQ MTX	49	43 (88)	0	3 (6)	0 (0)	2 (4)	1 (2)	0 (0)
Oral MTX	115	89 (77)	2 (2)	9 (8)	11 (10)	1 (1)	3 (2)	0 (0)
*P* value for row mean score < 0.0001								
*P* value for row mean score differs (excluding “No” responses) = 0.50								
		Severity of vomiting						
Therapy, *n* (%)	*N*	No	Trivial	Mild	Moderate	Marked	Severe	So severe I had to stop
Biologic therapies	218	210 (96)	0	5 (2)	1 (.5)	1 (.5)	1 (.5)	0 (0)
SQ MTX	49	45 (91)	2 (4)	1 (2)	0	1 (2)	0 (0)	0 (0)
Oral MTX	115	107 (93)	1 (1)	2 (1)	4 (4)	1 (1)	0 (0)	0 (0)
*P* value for row mean score = 0.63								
*P* value for row mean score differs (excluding “No” responses) = 0.15								
		Severity of nausea						
Therapy, *n* (%)	*N*	No	Trivial	Mild	Moderate	Marked	Severe	So severe I had to stop
Biologic therapies	218	190 (87)	0 (0)	7 (3)	11 (5)	6 (3)	2 (1)	2 (1)
SQ MTX	49	22 (45)	0 (0)	7 (14)	11 (22)	4 (8)	3 (6)	2 (4)
Oral MTX	115	76 (66)	12 (10)	17 (15)	10 (9)	0 (0)	0 (0)	0 (0)
*P* value for row mean score < 0.0001								
*P* value for row mean score differs (excluding “No” responses) = 0.21								
		Severity of other stomach problems						
Therapy, *n* (%)	*N*	No	Trivial	Mild	Moderate	Marked	Severe	So severe I had to stop
Biologic therapies	218	204 (94)	0 (0)	3 (1)	4 (2)	5 (2)	2 (1)	0 (0)
SQ MTX	49	46 (94)	0 (0)	1 (2)	0	0	2 (4)	0 (0)
Oral MTX	115	108 (94)	0 (0)	1 (1)	5 (4)	0	1 (1)	0 (0)
*P* value for row mean score = 0.87								
*P* value for row mean score differs (excluding “No” responses) = 0.50								
		Severity of fatigue						
Therapy, *n* (%)	*N*	No	Trivial	Mild	Moderate	Marked	Severe	So severe I had to stop
Biologic therapies	218	170 (78)	1 (.5)	5 (2)	17 (8)	10 (4.5)	13 (6)	2 (1)
SQ MTX	49	13 (27)	0	4 (8)	16 (33)	5 (10)	10 (20)	1 (2)
Oral MTX	115	51 (44)	0	5 (4)	25 (22)	23 (20)	10 (9)	1 (1)
*P* value for row mean score = <0.0001								
*P* value for row mean score differs (excluding “No” responses) = 0.90								
		Severity of malaise						
Therapy, *n* (%)	*N*	No	Trivial	Mild	Moderate	Marked	Severe	So severe I had to stop
Biologic therapies	218	187 (86)	2 (1)	3 (1)	13 (6)	9 (4)	3 (1)	1 (1)
SQ MTX	49	26 (53)	1 (2)	4 (8)	12 (24)	5 (10)	1 (2)	0 (0)
Oral MTX	115	77 (67)	0	12 (10)	20 (17)	5 (4)	1 (1)	0 (0)
*P* value for row mean score = <0.0001								
*P* value for row mean score differs (excluding “No” responses) = 0.09								
		Severity of mental fog						
Therapy, *n* (%)	*N*	No	Trivial	Mild	Moderate	Marked	Severe	So severe I had to stop
Biologic therapies	218	185 (85)	0	7 (3)	15 (7)	7 (3)	2 (1)	2 (1)
SQ MTX	49	31 (63)	0	4 (8)	11 (22)	3 (6)	0	0 (0)
Oral MTX	115	82 (71)	2 (2)	15 (13)	9 (8)	6 (5)	1 (1)	0 (0)
*P* value for row mean score = 0.01								
*P* value for row mean score differs (excluding “No” responses) = 0.03								
		Severity of infection						
Therapy, *n* (%)	*N*	No	Trivial	Mild	Moderate	Marked	Severe	So severe I had to stop
Biologic therapies	218	195 (95)	0 (0)	3 (1)	8 (1)	3 (1)	5 (2)	4 (2)
SQ MTX	49	45 (92)	0 (0)	1 (2)	2 (4)	1 (2)	0 (0)	0 (0)
Oral MTX	115	110 (96)	0 (0)	1 (1)	3 (2)	1 (1)	0 (0)	0 (0)
*P* value for row mean score = 0.06								
*P* value for row mean score differs (excluding “No” responses) = 0.16

## Discussion

This study is among the few that assessed the frequency of patient symptoms and side effects associated with initiation of SQ and oral methotrexate in a real-world setting. We found several significant differences in the frequency of several of these compared to biologic therapy. For example, between one-third and one-half of patients receiving either formulation of MTX reported malaise and between half and three-quarters reported fatigue, a much greater proportion than in the biologic users (22 %). At least some hair loss was reported by approximately 30 % of patients in both MTX arms. The patient-reported prevalence of diarrhea was lower among patients receiving SQ MTX than among those receiving oral MTX, although nausea was more frequent with SQ MTX. Rates of mental fog and hair loss also were highest among patients receiving SQ MTX, and the greater prevalence of these symptoms may possibly reflect higher drug levels. Both the incidence and the magnitude of injection pain associated with SQ MTX was significantly lower than that associated with etanercept and adalimumab.

Results from clinical trials of RA patients initiating MTX generally find lower rates of MTX- and biologic-associated adverse events than found in our survey. For example, the TEAR trial found relatively low rates of AEs associated with MTX monotherapy as given to early RA patients for up to 2 years [19, 20], as did the COMET trial [21]. However, observational studies, which may have better generalizability for patients seen in routine care, offer more heterogeneous results on this topic. A single-center observational study of 248 U.S. patients found low rates of MTX discontinuation [4]. A U.K. study conducted in two hospitals found that among the patients changed from oral to SQ MTX, lack of efficacy (51 %) and adverse events (44 %) were similarly common, and high rates of subsequent persistence with SQ MTX were observed (75 % at 2 years) [22]. In contrast, larger studies have generally found lower adherence and poorer tolerability. For example, a large U.K. cohort of 1257 patients (predominantly RA) found that approximately 1/3 discontinued MTX, predominaly due to GI tolerability [5]. This result is consistent with findings from an older observational Norwegian study of 1648 patients that showed that 34 % of patients had discontinued at 2 years [23].

A number of studies have found better bioavailability with SQ MTX compared to oral MTX [8, 24], which may translate into improved efficacy among patients who switch from oral to SQ MTX [13, 25–27]. However, this effect may increase the rate of side effects associated with more bioavailable MTX. Although not specifically addressed in our survey, the adverse event profile associated with MTX also has been shown to be influenced by folate supplementation [28].

The frequency of pain associated with SQ injection was greater for biologic users (84 %) compared to SQ MTX users (55 %), as was the magnitude of pain. Injection pain might be further reduced by using an MTX auto-injector, which has been rated by patients as easy to use and nearly pain-free [10]. While patients generally did not have very high out-of-pocket co-payments for MTX, some patients receiving oral MTX expressed a higher willingness to pay if MTX might have greater effectiveness or an improved side effect profile.

The strengths of our study include representation of a patient population receiving care in routine settings. Prior findings based predominantly on clinical trials of biologics would presumably have much poorer generalizability to a real-world experience. Of importance in this study, patients needed to have started MTX or biologics in the last 12 months but had no requirement that they remain on these therapies. This design feature therefore avoided a potential selection bias if only prevalent and ongoing MTX and biologic users were eligible to participate. Were this the case, we presumably would have found a lower prevalence of various symptoms and side effects that might have prompted discontinuation. Additionally, we specifically prompted for various symptoms and AEs of interest, which patients may feel more comfortable with reporting in a survey, unlike clinical trials that passively ascertain such symptoms only if reported as an AE. We note that patients reported on the prevalence and severity of some symptoms (e.g. fatigue) that could either be drug related, drug dose related (for MTX), and/or disease related. Thus, the lower incidence of these symptoms in biologic treated patients could reflect either effects of the drugs, a lower median dose of MTX being used, or more effective control of disease-associated symptoms. We also recognize that in the analysis of MTX dose and associated symptoms, confounding by disease severity may have influenced our results. However, the comparison with the results of the patients on biologic therapy would somewhat argue against this, given that these patients presumably had more severe disease that warranted biologic use.

Despite these strengths, several limitations of this analysis deserve mention. The survey population was a convenience sample, and people who are members of an online patient community may be different than those seen in routine medical care settings. For example, they may have more severe, or more symptomatic RA, that prompts them to seek help online. Also, patients had self-reported RA, and while the positive predictive value (PPV) of patient-reported RA is relatively low, the PPV of patient reported RA among those who also report DMARD or biologic use is appreciably higher [29]. We acknowledge that medication use was self-reported and was not externally confirmed. Importantly, our survey was cross-sectional and patients’ responses were potentially subject to recall bias. We also did not collect information about comorbidities or RA disease activity, since with a cross-sectional design, it would not be possible to disentangle the relative contribution of these factors to the outcomes we studied. Also, were not able to assess improvement or worsening in patients who changed medications. For example, the differential outcomes of RA patients who experienced GI side effects (e.g. severe nausea) while on oral MTX who then subsequently changed to SQ MTX (who then experienced less nausea) could not be ascertained since longitudinal data would be required. Generating this type of data will be useful to better characterize the benefits of changing from oral MTX to SQ MTX or biologics in future studies. Additionally, 29 % of patients in the SQ MTX arm had already discontinued, a much higher proportion than in the oral MTX arm (8 %), suggesting some potential bias in the sample with respect to the prevalence of side effects that could have prompted discontinuation. We also recognize that most RA patients seldom start on SQ MTX initially but rather ‘fail’ oral MTX and then switch, suggesting the likelihood that more ill patients who need greater treatment efficacy, or those with a higher burden of GI side effects from oral therapy, probably were channeled to SQ MTX. We also recognize that etanercept and adalimumab-related injection site pain may differ according to whether patients were using the pre-filled syringe, the autoinjector pen, or the reconstituted lyophilized powder (for etanercept). The survey did not collect this information, and was therefore not able to differentiate between these different formulations. The survey also did not ask about folate use, which has the potential to mitigate MTX-associated side effects [30–32]. Finally, although we examined a comparator of patients treated with biologics, two-thirds of them were receiving concomitant MTX and their symptom profile likely reflected the contribution of both biologic and MTX exposure. However, these subgroups were separated in relevant analyses (e.g. Table 3).

## Conclusion

In conclusion, our results indicate that the prevalence of MTX and biologic-associated symptoms and side effects may be appreciably higher than prescribing information for these agents or anecdotal experience might suggest. Future longitudinal studies need to consider opportunities to optimize use of MTX and evaluate the potential benefit of switching from oral to SQ MTX in cases of intolerability or inefficacy, which may delay patients’ progression to biologic therapies, which may result in substantial cost savings [33]. Further work to characterize strategies to mitigate side effects associated with RA therapies (e.g. higher doses of folate or folinic acid) may be useful to maximize the benefits of MTX and other arthritis medications.

## Abbreviations

AEs: Adverse events
GI: Gastrointestinal
MTX: Methotrexate
RA: Rheumatoid arthritis
SD: Standard deviation
SQ: Subcutaneous
